# Deep learning to predict future cognitive decline: a multimodal approach using brain MRI and clinical data

**DOI:** 10.3389/fnimg.2026.1726037

**Published:** 2026-02-04

**Authors:** Tamoghna Chattopadhyay, Pavithra Senthilkumar, Rahul H. Ankarath, Christopher Patterson, Emma J. Gleave, Sophia I. Thomopoulos, Heng Huang, Li Shen, Lei You, Degui Zhi, Paul M. Thompson

**Affiliations:** 1 Imaging Genetics Center, Mark and Mary Stevens Neuroimaging and Informatics Institute, Keck School of Medicine, University of Southern California, Marina del Rey, CA, United States; 2 Department of Computer Science, University of Maryland, College Park, MD, United States; 3 Department of Biostatistics, Epidemiology and Informatics, Perelman School of Medicine, University of Pennsylvania, Philadelphia, PA, United States; 4 McWilliams School of Biomedical Informatics, University of Texas Health Science Center at Houston, Houston, TX, United States

**Keywords:** Alzheimer’s disease prognostics, artificial intelligence in hospitals, AutoGluon, clinical decision support systems, clinical decline, clinical dementia rating, deep learning, multimodal analysis

## Abstract

Predicting the trajectory of clinical decline in aging individuals is a pressing challenge, especially for people with mild cognitive impairment, Alzheimer’s disease, Parkinson’s disease, or vascular dementia. Accurate predictions can guide treatment decisions, identify risk factors, and optimize clinical trials. In this study, we compared two deep learning approaches for forecasting changes, over a 2-year interval, in the Clinical Dementia Rating scale ‘sum of boxes’ score (sobCDR), as a continuous outcome (regression). This is a key metric in dementia research and clinical trials, and scores range from 0 (no impairment) to 18 (severe impairment). To predict decline, we trained a hybrid convolutional neural network (CNN) that integrates 3D T1-weighted brain MRI scans with tabular clinical and demographic features (including age, sex, body mass index (BMI), and baseline sobCDR). We benchmarked its performance against AutoGluon, an automated multimodal machine learning framework that selects an appropriate neural network architecture (an ‘autoML’ approach). We evaluated the models using data from 2,319 unique participants drawn from three independent cohorts—ADNI, OASIS-3, and NACC. For each participant, we used one T1-weighted brain MRI scan along with corresponding clinical and demographic information. Our results demonstrate the importance of combining image and tabular data in predictive modeling for this clinical application. Deep learning algorithms can fuse information from image-based brain signatures and tabular clinical data, with potential for personalized prognostics in aging and dementia. Rather than concluding that multimodal fusion uniformly improves performance, our results show that deep learning applied to volumetric MRI data may struggle to add predictive value, particularly when clinical covariates explain substantial variance and provide a strong baseline. In other conditions and tasks, it may help to have a hybrid system that can learn from both data types, and their relative value may be different. Conversely, AutoML-based multimodal fusion provides a robust baseline when tabular data already provide strong predictive value for the task. These insights clarify how different multimodal strategies could be selected in clinical prognostic applications.

## 1 Introduction

Predicting future clinical decline in individuals with mild cognitive impairment (MCI) or preclinical stages of other neurodegenerative diseases is critical for early diagnosis, treatment planning, and therapeutic intervention. Neurodegenerative conditions, including Alzheimer’s disease (AD), Parkinson’s disease, and vascular dementia, affect millions worldwide and impose significant personal, societal, and economic costs. Among individuals diagnosed with MCI, approximately 10–15% progress to dementia annually, a transition marked by a subtle yet measurable worsening of clinical symptoms (World Health Organization, 2017). The Clinical Dementia Rating (CDR) scale (Cedarbaum et al., 2013), particularly the sum of boxes score (sobCDR), is a widely-used measure of the severity of cognitive and functional impairment– rating impairment on a scale of 0 (none) to 18 (severe). Accurate prediction of future sobCDR scores is essential for assessing disease progression, guiding clinical trial design, and tailoring interventions to patient-specific trajectories.

**Figure 1:**
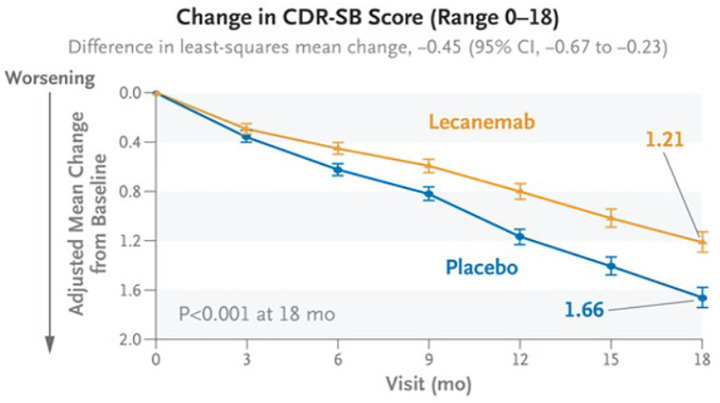
Progressive decline in sobCDR score is a primary outcome measure in many dementia drug trials (here, in a clinical trial evaluating the anti-amyloid drug, lecanemab). Reproduced from Van Dyck et al. (2023).

Predicting clinical decline is challenging, as multiple risk factors affect the onset and progression of neurodegenerative diseases. Cognitive and functional impairments in aging individuals are influenced by genetic, environmental, and lifestyle factors. Abnormal brain changes, observable with neuroimaging, may precede clinical symptoms by years or even decades. Predictive models must account for this broad range of causal or influential factors by integrating multimodal data from neuroimaging, demographics, clinical history, and baseline clinical measures. However, the heterogeneity of these data types poses challenges for many traditional machine learning algorithms, which may need to merge information from structured and unstructured inputs. If successful, accurate prognostic models could also be used to gauge treatment effects in clinical trials. As Figure 1 shows, recent advancements in therapeutic interventions, such as the anti-amyloid drug, lecanemab (Van Dyck et al., 2023), can significantly reduce the rate of decline as measured by sobCDR; this randomized clinical trial found a 27% slowing after 18 months. Treatment effects would be easier to identify if statistical models could more accurately identify factors that influence disease trajectories based on baseline clinical and neuroimaging data.

Table 1 provides a structured summary of key prior studies on predicting clinical decline in dementia, detailing the datasets used, methodological approaches, reported performance, identified limitations, and the overall impact of each study. Prior work predicting clinical decline from multimodal data includes the TADPOLE challenge (Marinescu et al., 2019), in which 33 teams used 92 algorithms to predict AD progression from multimodal biomarkers. Even so, TADPOLE used only one dataset (ADNI), making it unclear how well performance might generalize to other cohorts. Recent work has also leveraged a variety of machine learning and deep learning approaches (Delor et al., 2013; Williams et al., 2013; Dhinagar, 2023, 2024, 2025; Odusami et al., 2023; Hoang et al., 2023; Zarei et al., 2024; Hebling Vieira et al., 2022; Bapat et al., 2024; Kale et al., 2024). Traditional statistical models, such as linear regression (Mattsson-Carlgren et al., 2023; Dickerson et al., 2007) and Cox proportional hazards models (Wang et al., 2022; Rongve et al., 2016; Kim et al., 2019), can be used to predict clinical decline but are not well-suited to including entire images as inputs, or fusing multimodal data streams. More recent innovations include ‘Longitudinal-to-Cross-sectional’ (L2C) transformations to convert variable longitudinal histories into fixed-length feature vectors; other researchers have fitted models to entire longitudinal histories, as in the ‘AD Course Map’ (AD-Map) approaches and methods using minimal recurrent neural networks (MinimalRNN) (Zhang et al., 2025). More recently, computer vision-based deep learning architectures have been adapted for biomedical image analysis to extract diagnostic or prognostic features, and identify biomarkers of neurodegenerative diseases. For example, convolutional neural networks (CNNs) and vision transformers have been applied to analyze 3D T1-weighted MRI scans, yielding insights into structural brain alterations associated with AD (Chattopadhyay et al., 2024a; Chattopadhyay et al., 2024b). AutoML platforms, such as AutoGluon (Erickson et al., 2020), have also gained traction as they can optimize and ensemble models across diverse datasets, or select neural network architectures that perform best for a task– achieving competitive performance with minimal manual intervention.

**Table 1:** Summary of key studies on predicting clinical decline in dementia: datasets, methods, performance, limitations, and impact.

Authors/teams	Year	Dataset(s)	Methods Used	Performance/Outcome	Limitations	Significance
Marinescu et al. (2019) (TADPOLE Challenge)	2019	ADNI	92 ML/DL algorithms, multimodal	MAUC up to 0.931 (diagnosis), ~0.41 MAE (ventricle vol)	Only ADNI dataset, unclear generalizability	Open benchmark for AD prediction, multimodal data shown to help
Prosser et al. (2023)	2023	ADNI2/GO, ADNI3	Cox regression (WMH, CSF, MRI)	C-index 0.77–0.81; WMH & hippocampal vol predicted CN → MCI; CSF and MRI most predictive MCI → AD	Single-site population health, only baseline data, does not use deep learning	Combined classical & imaging biomarkers can robustly predict progression
Grueso and Viejo-Sobera (2021) (systematic review)	2021	Multiple (mainly ADNI)	SVM, CNN, multimodal ML/DL	SVM: ~75% accuracy, CNN: ~78%, multimodal best; metrics vary	Heterogeneity in reporting, comparability between studies	Multimodal DL is state-of-the-art for clinical decline prediction
Wang et al. (2022)	2022	Shanghai Aging cohort	Cox regression, ML (random forest)	ML AUC 0.81, Cox C-index 0.65–0.77	Single-population, mostly tabular with limited imaging	Demonstrates cox vs. non-linear ML for MCI → AD risk
Mattsson-Carlgren et al. (2023)	2023	Swedish BioFINDER	Cox model, deep learning, plasma biomarkers	Cox C-index up to 0.83; plasma + PET best	Limited to preclinical, biofluid biomarker focus	Plasma biomarkers have measurable predictive value
Ashish and Turner (2024)	2024	ADNI	Feature-based supervised ML	Clinical+demo or neuroimage models: clinical alone can be highly predictive	Imaging not always best, integration needed, ADNI-based	Predictive power from non-imaging clinical variables nearly as strong as imaging
Thulasimani et al. (2024) (review)	2024	Many (ADNI, AIBL, OASIS, etc.)	Deep learning (CNN, RNN, GAN), ML	Accuracies 73–99% (varies by model/dataset/task)	Small datasets, heterogeneity, EEG not always available	Multimodal, many methods reviewed, emerging translational techniques
Zhang et al. (2025) (cross-dataset evaluation)	2024	ADNI, OASIS, NACC	MinimalRNN, L2C deep longitudinal	R^2^ 0.6–0.72 for best methods	Validation not always possible on all endpoints	Deep learning can generalize, but longitudinal design & test cohort selection matter
Bron et al. (2015) (CADDementia Challenge)	2015	CADDementia	SVM, ML ensemble	MAUC up to 0.78 on multi-class problem	Only cross-sectional, subtype (not longitudinal progression)	Early benchmark for multi-class diagnostic challenge
Jung et al. (2024)	2024	Korean SMC cohort	Attention RNN, multimodal (PET/MRI)	AUC: ~0.81, R^2^ ↑ with multimodal imaging	Single center, non-ADNI, requires PET	Shows multimodal deep learning can generalize on new ethnic cohorts
Chun et al. (2022)	2021	Korea, multiple hospitals	ML classifier on MMSE + clinicals	Sensitivity/Specificity ~80%/50%	Clinical data only, lacks biomarkers	Clinical tests alone more accessible, but less discriminative
Dickerson et al. (2007)	2007	Massachusetts General Hospital	Logistic regression, neuropsychological tests + CDR-SB	OR 4.8 per point increase in CDR-SB for AD prediction; predictive of decline over 5 years	Focus on clinical/psychometric data, not imaging-based	Highlights utility of combined clinical, cognitive testing for AD prognostics
Rongve et al. (2016)	2016	Norwegian cohort	Longitudinal cohort study	Cognitive decline measured over 5 years in dementia with Lewy bodies	Small cohort, disease-specific (DLB)	One of few longitudinal studies on DLB cognitive decline trajectory
Kim et al. (2019)	2019	Korea periodic health exams	Cox proportional hazards vs. DL	Both methods predicted dementia; DL showed superior accuracy (C-index/D-index higher)	Limited to periodic health exam data; cohort from Korea	Comparison of classic survival model vs. deep learning for dementia prognosis

This study evaluates a custom hybrid deep learning model that integrates tabular clinical variables with structural MRI image data, and compares its performance against the AutoGluon framework for predicting two-year changes in sobCDR scores. Our hybrid model uses a CNN to extract meaningful features from 3D T1-weighted brain MRI scans and combines these with fully connected layers that process tabular inputs, including demographic and clinical data. AutoGluon (Erickson et al., 2020)–an automated machine learning framework–serves as a robust baseline for comparison. This work provides a systematic benchmarking comparison of two distinct multimodal strategies for predicting longitudinal clinical decline: (i) a full 3D volumetric fusion model that jointly learns from MRI and tabular data, and (ii) a multimodal AutoML strategy (AutoGluon) that integrates tabular features with reduced 2D imaging representations. Rather than assuming that adding imaging improves performance, we analyze how data modality dominance, imaging dimensionality, and sample size shape multimodal model behavior. Our results demonstrate that AutoML fusion can outperform volumetric CNN fusion when tabular features are strong predictors of clinical trajectories, whereas volumetric fusion likely requires richer imaging or larger training cohorts to leverage structural brain biomarkers, providing actionable guidance for selecting prognostic models in aging and dementia. By addressing these aspects, our work lays the groundwork for improving prognostic modeling in neurodegenerative diseases and fostering the development of personalized predictive models. The rest of this paper is structured as follows: Section 2 describes the datasets, preprocessing, and model architectures; Section 3 presents experimental results and comparisons; Section 4 discusses the implications of the experimental results; and Section 5 concludes with limitations of the study and future directions. Importantly, we do not assume that adding MRI imaging necessarily improves prediction accuracy. Instead, we investigate how multimodal models behave in realistic clinical scenarios, such as having highly informative baseline covariates (e.g., sobCDR), limited sample size relative to input dimensionality, and reduced 2D imaging inputs. This allows us to identify when volumetric MRI adds prognostic value and when simpler strategies may be preferable. A preliminary version of this work was released as a paper at IEEE SIPAIM 2025, where an early hybrid CNN and a single AutoGluon variant were presented without detailed benchmarking or statistical evaluation. In contrast, the current manuscript (i) systematically compares multiple 2D and full 3D multimodal configurations, and (ii) introduces statistical hypothesis testing to assess performance differences. Collectively, these additions expand the scope for this paper.

## 2 Materials and methods

**Figure 2:**
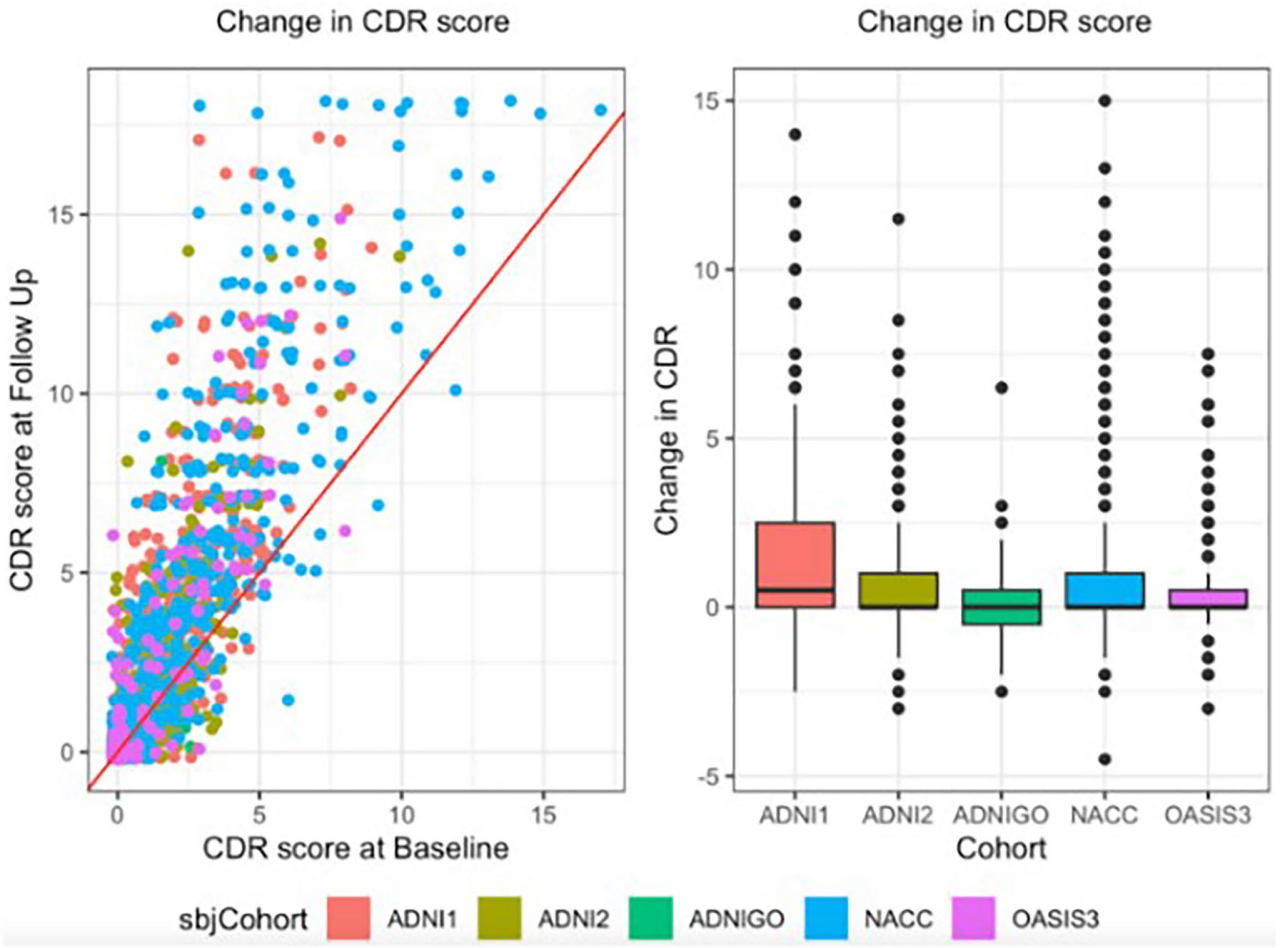
Individuals from different cohorts are shown in different colors; the ADNI dataset is divided into 3 components collected during different phases of the study (1, 2, and ‘GO’). Changes in sum-of-boxes score (sobCDR) over 2 years for individual participants in each dataset are shown in the scattergram (left) and box-and-whiskers plots (right), split by cohort. Higher scores denote greater impairment.

To evaluate the proposed predictive models, we used data from three independent, publicly available cohorts: (1) the Alzheimer’s Disease Neuroimaging Initiative (Jack Jr et al., 2008) (ADNI; 1,136 participants: 73.72 ± 7.11 years, 507 F/629 M; 397 cognitively normal (CN), 593 MCI, 146 AD), (2) the Open Access Series of Imaging Studies (LaMontagne et al., 2019) (OASIS-3; 241 participants: 72.88 ± 6.44 years, 113 F/128 M; 172 CN/ 62 AD), and (3) the National Alzheimer’s Coordinating Center (Beekly et al., 2007) (NACC; 942 participants: 72.23 ± 9.38 years, 413 F/529 M; 546 CN, 211 MCI, 185 AD). We included participants who met the following inclusion criteria: availability of a 3D T1-weighted brain MRI at baseline, baseline measurements of age, sex, and body mass index (BMI), a baseline sum of boxes Clinical Dementia Rating (sobCDR) score recorded within 90 days of the imaging session, and a follow-up sobCDR score measured between 1.75 and 2.25 years after the baseline visit. These criteria ensured that all participants had comparable data for longitudinal analysis of clinical decline. Figure 2 gives descriptive statistics which summarizes the overall trends in the training and test data.

**Figure 3:**
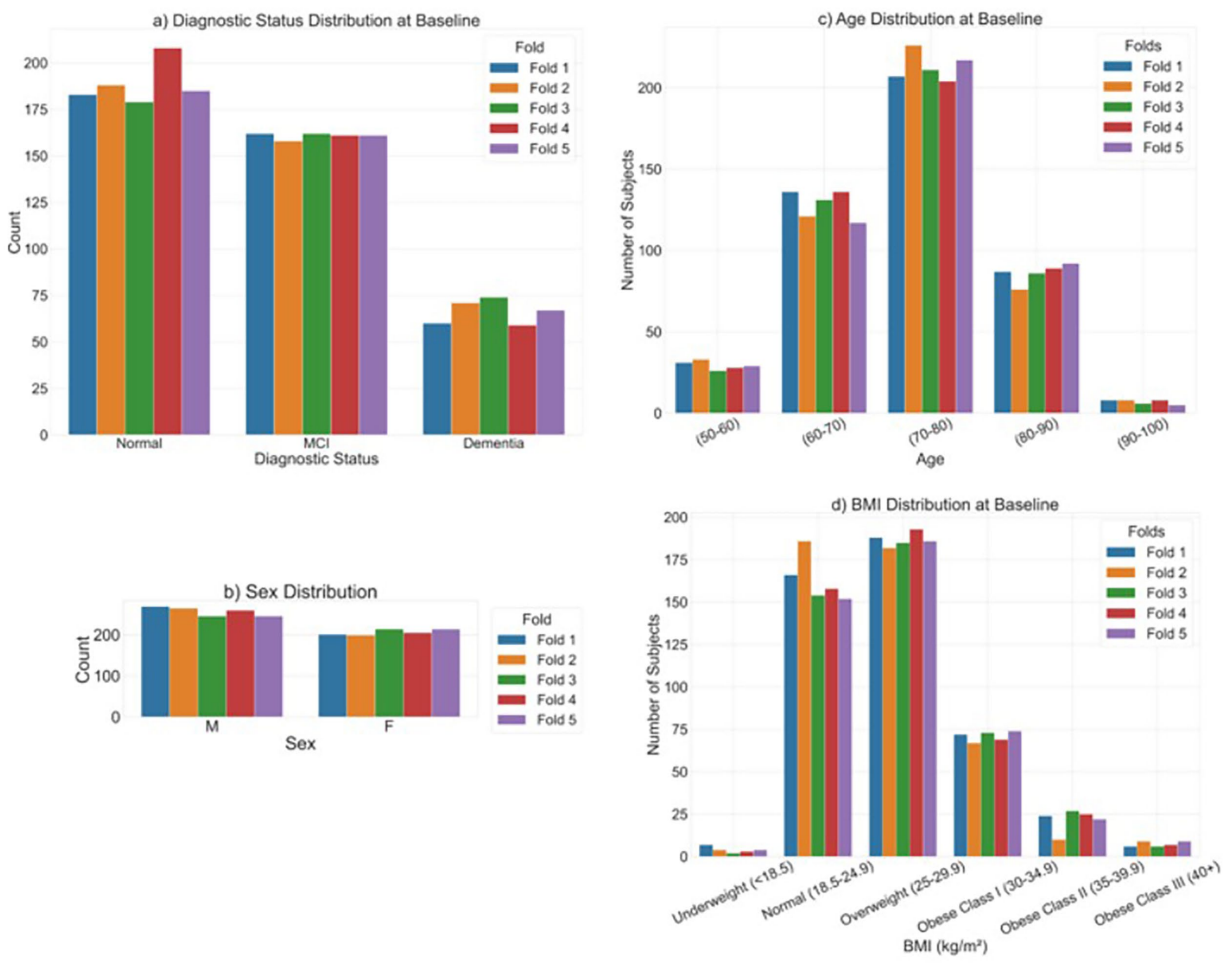
**(a)** Distribution of diagnostic status at baseline, across the five folds used for cross-validation. We also show the distribution of **(b)** Sex, **(c)** Age at baseline, and **(d)** BMI at baseline, across the five folds.

For evaluation, datasets were partitioned into five independent cross-validation folds (Bates et al., 2024), stratified to ensure balanced distributions of sex, baseline age at imaging, and changes in sobCDR scores. This stratification was crucial to mitigate potential biases and to ensure that each fold represented a representative subset of the data, with changes in sobCDR ranging from 0 (no impairment) to 18 (severe impairment). This is shown in Figure 3.

A preprocessing pipeline was applied to all imaging data to standardize and enhance the quality of the input features. 3D T1-weighted brain MRI volumes were pre-processed using the following steps: nonparametric intensity normalization [N4 bias field correction using ANTS (Isensee et al., 2019)], ‘skull stripping’ for brain extraction using HD-BET (Avants et al., 2008; Tustison et al., 2010), nonlinear registration to a template with 6 degrees of freedom and isometric voxel resampling to 2 mm (Chattopadhyay, 2023; Lam et al., 2020). Although higher-resolution or patch-based models might capture finer local anatomical detail, such approaches require additional GPU capacity and typically restrict the effective training sample size for deep multimodal models. In prognostic tasks, reducing the sample size would be expected to negatively impact generalization more than marginal voxel-level resolution gains would improve prediction accuracy. Patch- or crop-based learning also discards broader structural context, which can help for modeling distributed neurodegeneration. Therefore, 2 mm resampling represents a practical tradeoff that enables stable and reproducible training while preserving the whole-brain features required for prognostic modeling. Pre-processed images were of size 91x109x91. The T1w images were scaled, via min-max scaling, to the range 0 and 1. All T1w images were aligned to a common template provided by the ENIGMA consortium (Jahanshad et al., 2013). Preprocessing decisions may influence predictive performance. For example, choice of registration template and interpolation method may affect preservation of fine structural details, and the degree of skull-stripping accuracy may influence downstream feature extraction. While our pipeline used widely adopted ENIGMA templates and trilinear resampling, future work will examine alternative harmonization strategies and their impact on model robustness.

Although harmonization (Komandur, 2023; Liu et al., 2023; Moyer et al., 2020; Sinha, 2021) tools such as ComBat, ComBat-GAM, or neuroHarmonize are widely used in cross-sectional classification studies, applying them in prognostic regression tasks requires caution. These methods typically adjust MRI based on covariates (e.g., age, diagnosis, or severity), which can theoretically introduce outcome leakage or suppress disease-related longitudinal variance when the prediction target is a change score (*Δ* sobCDR). In addition, harmonizing only the imaging modality may disrupt consistency with clinical variables during multimodal fusion, confounding the benchmarking objective of this study. Therefore, to isolate modeling differences between 3D volumetric fusion and AutoML fusion, no inter-site harmonization was applied in this work, and site heterogeneity is reported as a limitation rather than removed.

**Figure 4:**
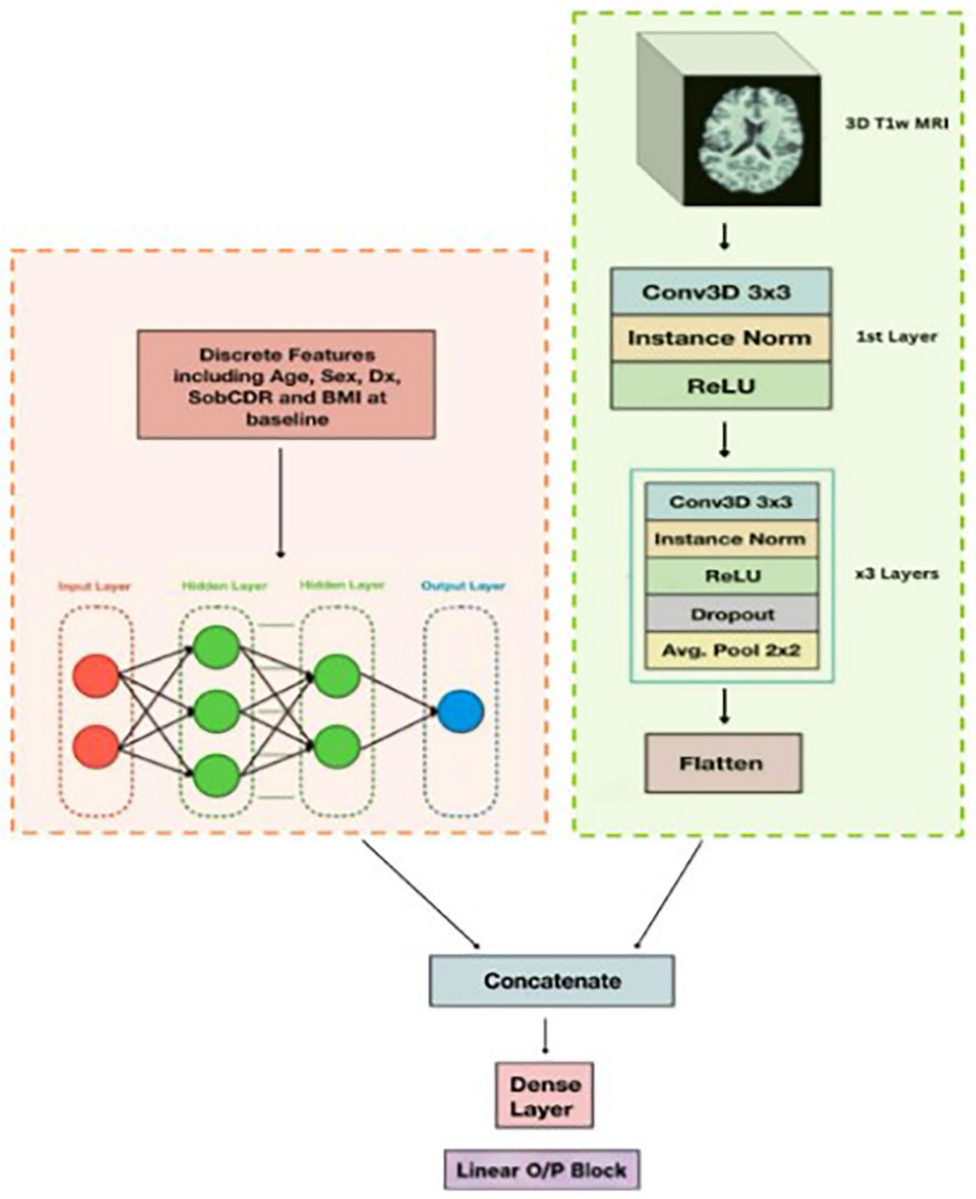
Hybrid 3D CNN model architecture. The Y-shaped design merges discrete features in tabular format (left) with entire 3D images (right), which are handled with a 3D CNN. The entire model is trained end-to-end.

Our proposed hybrid model (Chattopadhyay et al., 2024c) fuses a 3D convolutional neural network (CNN) with an artificial neural network (ANN), in a Y-shaped architecture (Figure 4) to combine imaging and tabular data for predicting clinical decline. The 3D CNN processes 3D volumetric T1-weighted MRI scans, while the ANN ingests discrete tabular features, including age, sex, BMI, and baseline sobCDR. These two data modalities are initially processed independently, before being fused for final prediction. The network is trained end to end.

The 3D CNN component (Figure 4) has three convolutional blocks, each incorporating filters of increasing size (32, 64, 128, and 256) to progressively extract hierarchical features from the MRI scans. Each convolutional block is followed by Batch Normalization and Max Pooling layers to enhance training stability and spatial feature reduction. The final convolutional layer, before concatenation, uses a filter size of 256 and employs average pooling to retain salient global spatial features while minimizing dimensionality. In parallel, the ANN component processes the tabular data using three fully connected layers with hidden sizes of 1,024, 512, and 64, respectively. Each layer is accompanied by instance normalization and the ReLU activation function to ensure stable and efficient learning. The structured numerical and categorical variables are first normalized and embedded appropriately, before being passed through the ANN. After processing through their respective architectures, the feature representations from the CNN and ANN components are concatenated via tensor stacking. The combined feature map is then passed through two fully connected dense layers with 128 and 64 units, respectively, each followed by ReLU activation and dropout (*p* = 0.5) to reduce overfitting. A final dense layer with a single linear output unit produces the predicted sobCDR score at 2 years. The model was trained with a learning rate of 0.001 using the Adam optimizer, with a batch size of 2 to handle the computational demands of 3D imaging data. Training was conducted over 200 epochs, ensuring sufficient convergence, by stopping training when the validation loss did not decrease further over 10 epochs, while preventing overfitting. The model’s generalizability was validated using five-fold cross-validation, where the hybrid model was independently trained on four folds, and tested on the held out fifth sector of the data. Performance was assessed using mean absolute error (MAE) and the coefficient of determination (R^2^ score). The MAE and R^2^ values were recorded for each cross-validation fold, with the final performance determined as the average of these metrics across all five folds. MAE provides a clinically interpretable measure of prediction error in sobCDR units, while R^2^ quantifies the proportion of variance explained. Although RMSE is sensitive to extreme errors, we emphasize that such outliers in dementia progression may reflect atypical clinical courses or instrument variability rather than systematic model bias. For these reasons, MAE and R^2^ were chosen as the primary evaluation metrics in this study. We have also reported the Root Mean Square Error (RMSE), Median Absolute Error, Mean Absolute Scaled Error and the Symmetric Mean Absolute Percentage Errors. This ensured robust assessment of the model’s ability to predict longitudinal cognitive decline.

**Figure 5:**
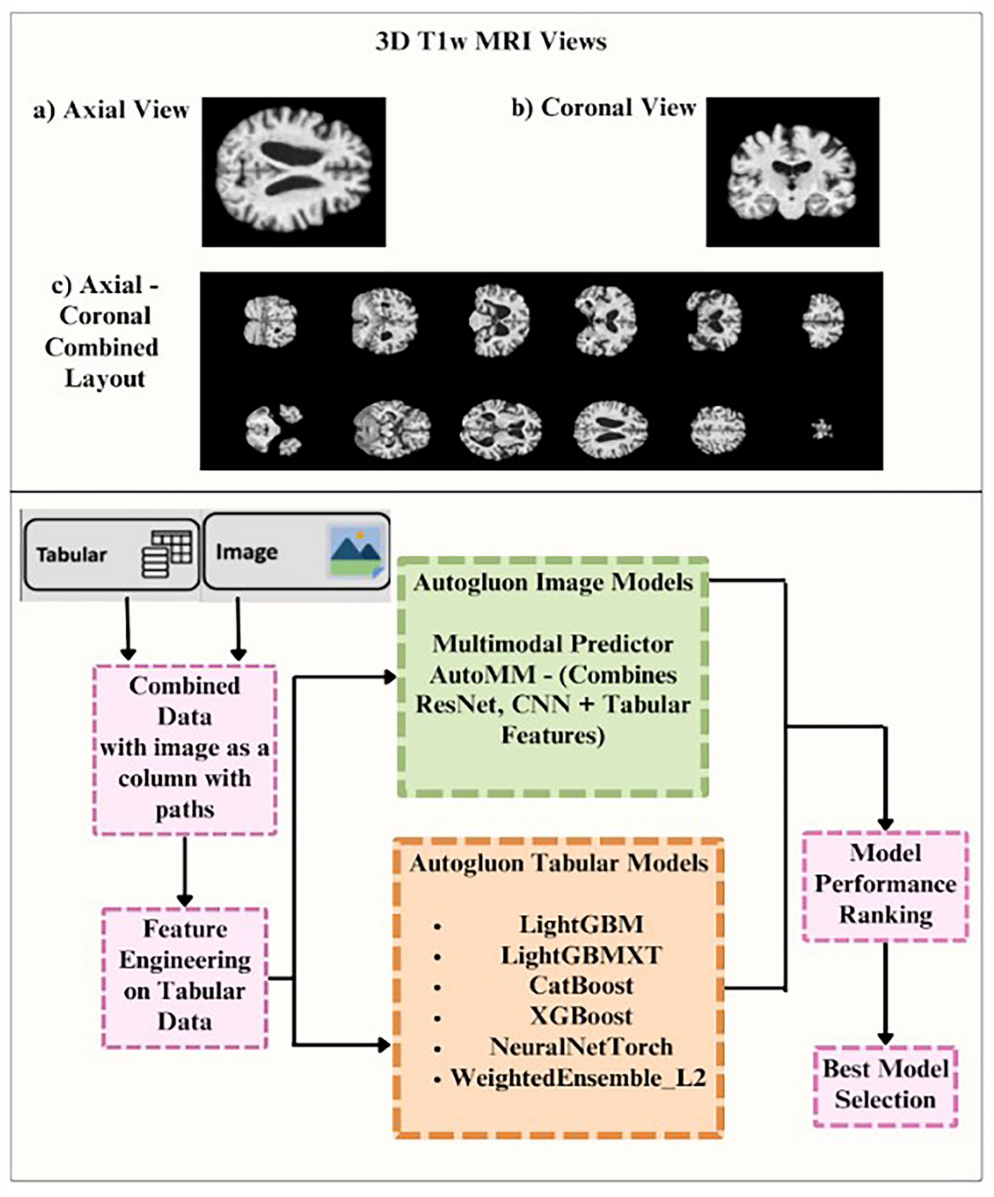
Top: image inputs for the AutoGluon models. Bottom: AutoGluon architecture, which automatically selects a neural network architecture from a range of traditional models.

To provide a comparative benchmark, we used AutoGluon (Erickson et al., 2020), an automated machine learning (AutoML) framework (Figure 5), which automatically selects a neural network architecture from a diverse set of traditional models, and trains it to predict future sobCDR scores. AutoGluon is well-suited for multimodal learning tasks, as it supports tabular, text, and image-based inputs with minimal user intervention. For the image input, rather than using full 3D brain MRIs, we extracted the 2D axial and coronal middle slices from the T1-weighted brain images. This approach reduces computational complexity while preserving structural information from relevant regions of the brain. While our hybrid CNN processes entire 3D brain volumes, AutoGluon was applied to 2D axial/coronal slices due to its lack of native support for 3D volumetric inputs. This dimensionality reduction simplifies computation but cannot support full volumetric representation (or 3D kernels). Thus, comparisons should be viewed as a benchmark against a widely used AutoML baseline, rather than a direct architectural equivalence. To partially address this limitation, we also ran experiments where multiple slices were selected and provided jointly to AutoGluon. Specifically, for each volume and orientation (axial and coronal), we first excluded invalid slices with minimal intensity variation (i.e., near-empty or non-brain regions). From the remaining valid slices, we then sampled a maximum of seven evenly spaced slices using the rule: step = max(1, total_slices // (max_slices + 2)), where total_slices corresponds to the number of valid slices along that axis and max_slices was set to 7. This ensured that the selected slices were distributed across the entire brain, capturing representative anatomical coverage while avoiding redundant or empty slices. The same discrete variables used for our hybrid deep learning model—age, sex, BMI, and baseline sobCDR—were included in the tabular input. AutoGluon automatically pre-processes the data, selects an appropriate neural network architecture, and tunes hyperparameters to optimize predictive performance. The training process involved automated selection of model ensembling strategies, ensuring robust predictions while reducing the risk of overfitting. We evaluated AutoGluon’s performance using the same five-fold cross-validation approach as our hybrid model, calculating average MAE and R^2^ scores across the folds. We have also reported the RMSE, Median Absolute Error, Mean Absolute Scaled Error and the Symmetric Mean Absolute Percentage Errors. To assess whether differences in predictive performance across models were statistically significant, paired t-tests were computed on fold-wise metrics obtained from the five-fold cross-validation. For each pairwise model comparison (e.g., Hybrid 3D CNN vs. Linear Regression; AutoGluon vs. Linear Regression), the mean absolute error (MAE) values from each fold were treated as paired observations. Two-sided paired t-tests were performed with *α* = 0.05 using the null hypothesis that both models produce equal mean MAE. The resulting *p*-values are reported in Table 2 and referenced in the Results and Discussion sections. This statistical approach evaluates whether observed model differences are consistent across folds, rather than driven by a single split or outlier cohort.

**Table 2:** Performance of deep learning architectures for predicting future clinical decline.

Model	Metric	Fold 1	Fold 2	Fold 3	Fold 4	Fold 5	Avg	*p*-value
*Linear Null Model*	MAE	0.980	1.027	1.13	0.998	1.012	1.096	
	R^2^	0.786	0.804	0.757	0.740	0.744	0.767	
*Hybrid CNN*	MAE	1.266	1.263	1.342	1.231	1.413	1.303	0.0013
	R^2^	0.763	0.658	0.694	0.716	0.687	0.704	0.0491
	RMSE	1.707	1.653	1.830	1.811	3.169	2.030	
	MedAE	0.440	0.547	0.478	0.437	2.512	0.883	
	MASE	0.385	0.390	0.386	0.385	0.962	0.501	
	sMAPE	118.126	113.609	114.767	122.189	121.518	118.042	
*AutoGluon axial sM*	MAE	0.946	0.964	1.083	1.057	0.958	1.002	0.2789
	R^2^	0.808	0.761	0.735	0.753	0.760	0.763	0.8357
	RMSE	1.692	1.631	1.823	1.714	1.602	1.692	
	MedAE	0.387	0.384	0.453	0.401	0.435	0.412	
	MASE	0.316	0.297	0.341	0.319	0.319	0.318	
	sMAPE	116.902	117.744	120.518	121.314	115.330	118.361	
*AutoGluon axial sR*	MAE	0.957	0.945	0.991	0.967	0.977	**0.968**	0.0469
	R^2^	0.794	0.767	0.745	0.777	0.772	**0.771**	0.7391
	RMSE	1.733	1.678	1.818	1.739	1.697	1.733	
	MedAE	0.327	0.368	0.352	0.402	0.429	0.376	
	MASE	0.312	0.304	0.323	0.326	0.332	0.319	
	sMAPE	119.457	119.629	117.128	120.434	117.531	118.836	
*AutoGluon axial mM*	MAE	1.048	1.072	1.051	0.964	0.976	1.022	0.8061
	R^2^	0.788	0.740	0.744	0.774	0.759	0.761	0.7698
	RMSE	2.548	3.780	3.062	3.030	3.635	3.211	
	MedAE	0.440	0.431	0.524	0.515	0.420	0.466	
	MASE	0.472	0.675	0.584	0.559	0.641	0.586	
	sMAPE	134.916	140.770	128.407	139.517	134.296	135.581	
*AutoGluon axial mR*	MAE	1.032	1.048	1.032	0.968	0.975	1.011	0.5153
	R^2^	0.754	0.753	0.750	0.765	0.761	0.757	0.5405
	RMSE	2.766	4.194	3.461	3.030	3.635	3.417	
	MedAE	0.357	0.400	0.457	0.515	0.420	0.430	
	MASE	0.486	0.735	0.639	0.559	0.641	0.612	
	sMAPE	138.034	143.782	134.152	139.517	134.296	137.956	
*AutoGluon coronal sM*	MAE	0.975	0.919	1.002	1.002	1.018	0.983	0.1929
	R^2^	0.787	0.768	0.726	0.752	0.760	0.759	0.5235
	RMSE	1.755	1.607	1.921	1.692	1.667	1.728	
	MedAE	0.364	0.346	0.305	0.401	0.449	0.373	
	MASE	0.323	0.296	0.328	0.326	0.341	0.323	
	sMAPE	117.690	119.190	119.180	119.353	112.865	117.655	
*AutoGluon coronal sR*	MAE	0.975	0.919	1.002	1.002	1.018	0.983	0.1929
	R^2^	0.787	0.768	0.726	0.752	0.760	0.759	0.5235
	RMSE	1.866	1.678	1.818	1.739	1.696	1.759	
	MedAE	0.307	0.367	0.352	0.402	0.428	**0.371**	
	MASE	0.327	0.303	0.323	0.326	0.332	0.322	
	sMAPE	122.430	119.629	117.128	120.434	117.531	119.430	
*AutoGluon coronal mM*	MAE	0.887	1.052	1.059	0.949	0.995	0.988	0.1192
	R^2^	0.793	0.728	0.722	0.757	0.744	0.748	0.3656
	RMSE	2.592	3.069	3.060	3.051	3.150	2.984	
	MedAE	0.446	0.355	0.489	0.454	0.515	0.451	
	MASE	0.483	0.563	0.606	0.569	0.597	0.563	
	sMAPE	135.032	137.018	139.409	138.782	127.164	135.481	
*AutoGluon coronal mR*	MAE	0.887	1.052	1.059	0.949	0.995	0.988	0.1192
	R^2^	0.793	0.728	0.722	0.757	0.744	0.748	0.3656
	RMSE	2.512	4.193	3.460	3.030	3.634	3.365	
	MedAE	0.540	0.400	0.457	0.514	0.420	0.466	
	MASE	0.497	0.734	0.639	0.559	0.641	0.614	
	sMAPE	141.163	143.781	134.151	139.516	134.296	138.581	
*AutoGluon AC sM*	MAE	0.976	0.902	1.033	1.021	1.028	0.992	0.2900
	R^2^	0.789	0.778	0.749	0.763	0.772	0.771	0.7084
	RMSE	1.429	1.656	1.818	1.745	1.492	**1.628**	
	MedAE	0.363	0.374	0.353	0.551	0.298	0.388	
	MASE	0.277	0.312	0.323	0.350	0.275	**0.307**	
	sMAPE	112.586	117.125	117.118	118.397	115.364	**116.11**8	
*AutoGluon AC sR*	MAE	0.941	0.968	0.992	1.017	0.986	0.981	0.1323
	R^2^	0.804	0.778	0.739	0.756	0.784	0.773	0.6499
	RMSE	1.771	1.678	1.624	1.740	1.492	1.661	
	MedAE	0.527	0.368	0.397	0.403	0.298	0.398	
	MASE	0.351	0.304	0.307	0.327	0.275	0.313	
	sMAPE	114.367	119.629	112.117	120.435	115.364	116.382	
*AutoGluon AC mM*	MAE	1.034	0.897	1.026	1.015	1.022	0.999	0.4466
	R^2^	0.728	0.779	0.750	0.764	0.774	0.759	0.6799
	RMSE	3.118	3.272	3.749	3.393	3.235	3.353	
	MedAE	0.449	0.417	0.627	0.449	0.390	0.466	
	MASE	0.598	0.579	0.730	0.639	0.577	0.625	
	sMAPE	129.826	128.559	143.987	145.431	137.977	137.156	
*AutoGluon AC mR*	MAE	0.996	0.961	0.987	1.012	0.981	0.987	0.2275
	R^2^	0.750	0.779	0.741	0.758	0.785	0.763	0.8142
	RMSE	3.069	4.194	3.351	2.846	3.235	3.339	
	MedAE	0.386	0.400	0.529	0.421	0.390	0.425	
	MASE	0.553	0.735	0.645	0.522	0.577	0.606	
	sMAPE	129.791	143.782	130.629	135.831	137.977	135.602	

For the AutoGluon models, axial means that the model used the middle axial slice as the image input, coronal means it used the middle coronal slice, and AC means it used multiple axial and coronal slices as the image input. S denotes standard scaling, m indicates MinMax scaling, M indicates MSE as the validation metric and R (used here as a suffix) indicates R^2^ as the validation metric. Best results are in bold. MedAE stands for median absolute error, MASE stands for mean absolute scaled error and sMAPE stands for symmetric mean absolute percentage error. *p*-values are calculated by paired T-tests between the linear null model and the other architectures, with an alpha of 0.05.

## 3 Results

**Figure 6:**
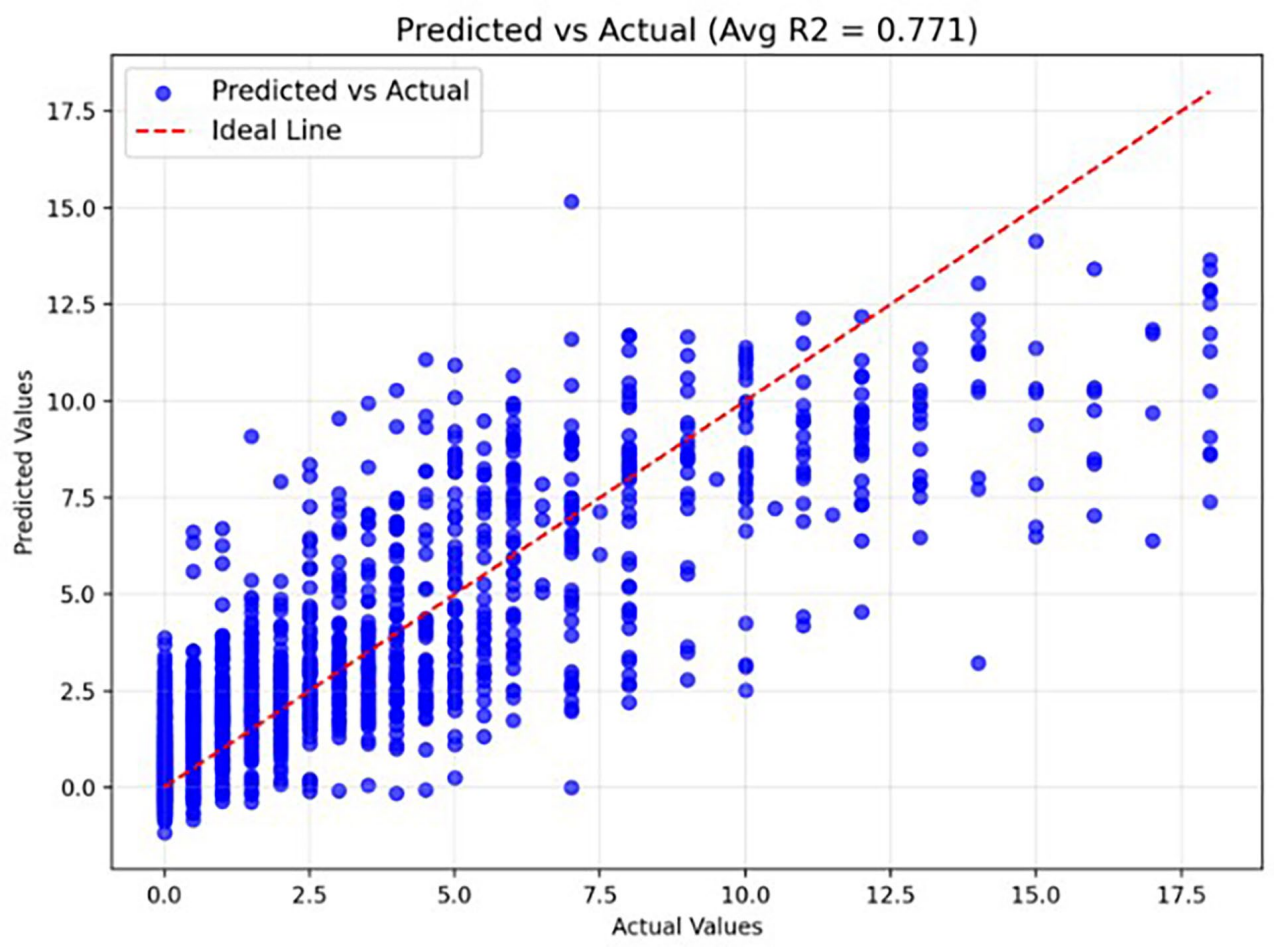
Actual vs. predicted scores (R^2^ score) when using the hybrid CNN architecture.

Our hybrid deep learning model was evaluated using five-fold cross-validation, with performance metrics recorded for each fold. The mean absolute error (MAE)–which measures the average deviation of predicted sobCDR scores from actual values–yielded an average of 1.303 across all folds, indicating strong predictive accuracy. In other words, the follow-up score is predicted with a mean error of just over 1 point, on an 18 point scale. The model’s coefficient of determination (R^2^), which quantifies the proportion of variance explained by the model, achieved an average of 0.704, demonstrating a strong correlation between predicted and actual clinical decline. Performance was consistent across folds: MAE values ranged from 1.231 to 1.414, and R^2^ values ranged from 0.659 to 0.763, highlighting the model’s robustness and reliability in forecasting cognitive decline over a two-year period. Figure 6 shows the Predicted vs. Actual values for a fold when using the hybrid CNN architecture. The Hybrid CNN did not consistently outperform the tabular-only Linear model baseline across folds, so further optimization of convolutional filter sizes, network depth, and fusion strategies may be needed to optimally use image features. Importantly, deep learning models are typically more data-hungry than linear baselines; thus, we expect that enlarging training cohorts with additional imaging and clinical data may further improve performance relative to the linear null model.

**Figure 7:**
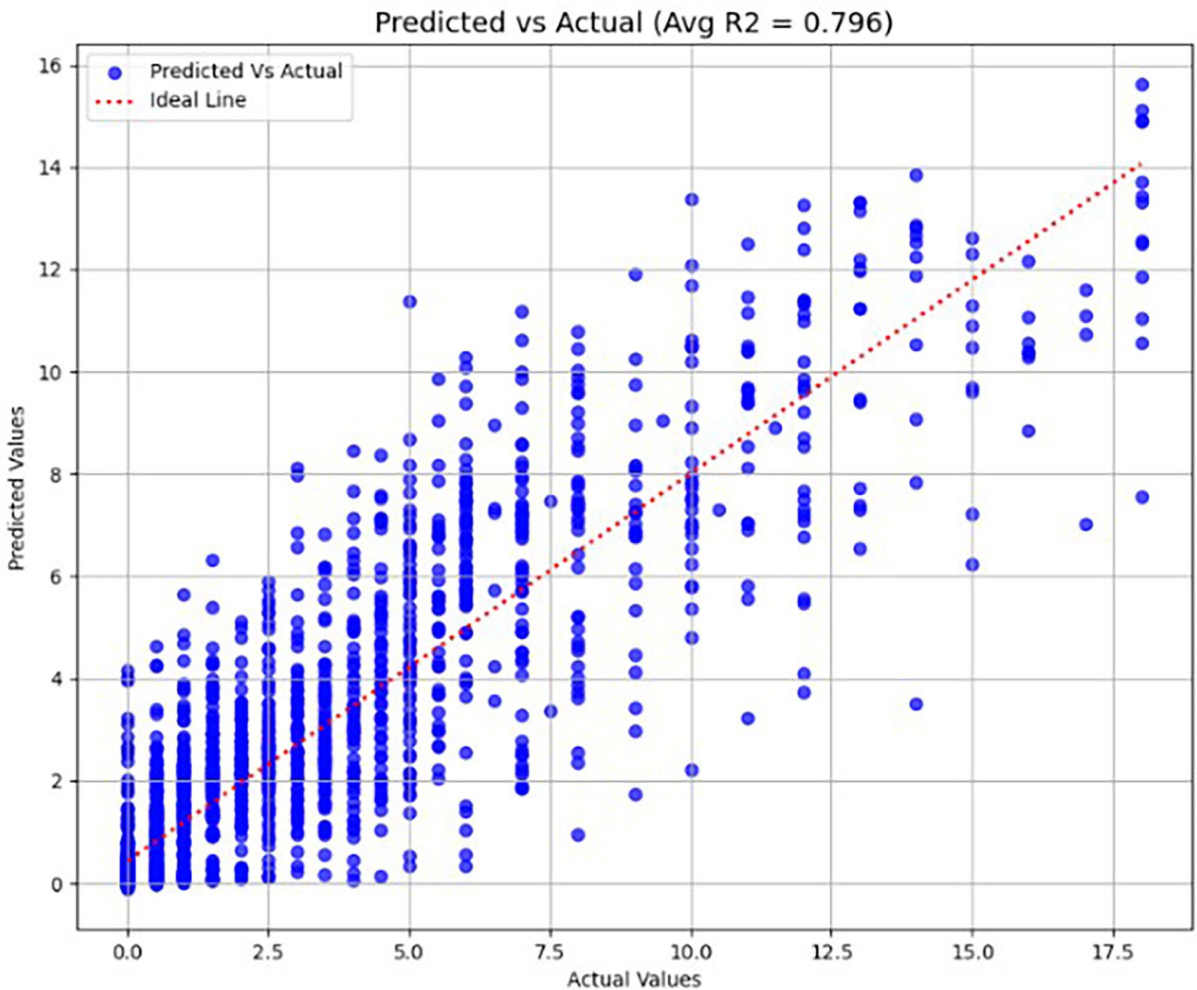
Actual vs. predicted scores (R^2^ score) when tested using AutoGluon for axial middle slice with standard scaled features and R^2^ validation.

We evaluated the performance of AutoGluon’s MultimodalFusionMLP model in predicting clinical decline using a combination of T1-weighted MRI middle axial slices and demographic features. The model was trained with approximately 97.4 M parameters using two different validation approaches and two scaling methods, for the tabular features, and assessed through 5-fold cross-validation. Using standard scaling, the model achieved an average R^2^ score of 0.763 and MAE of 1.002 when optimized for MSE validation. When optimized for R^2^ validation, the model showed similar performance with an average R^2^ of 0.771 and MAE of 0.968. With MinMax scaling, the model demonstrated comparable results. Under MSE validation, it achieved an average R^2^ score of 0.761 and MAE of 1.022. The R^2^ validation configuration yielded an average R^2^ of 0.757 and MAE of 1.011. Across all configurations, the model maintained consistent performance, with R^2^ scores ranging from 0.735 to 0.808, indicating robust predictive capability. The MAE values were stable across folds, typically between 0.94 and 1.08, suggesting reliable prediction accuracy regardless of the validation metric or scaling method chosen. The performance of AutoGluon’s MultimodalFusionMLP model when using a combination of T1-weighted MRI middle coronal slices and demographic features was slightly better. The same training configurations were used as the previous case - two different validation approaches and two different scaling methods, assessed through 5-fold cross validation. Using standard scaling, the model achieved an average R^2^ score of 0.759 and MAE of 0.983 across both validation models. Using min-max scaling, the model achieved an average R^2^ score of 0.749 and MAE of 0.988 across both validation models. Figure 7 shows the Predicted vs. Actual values for a fold when using AutoGluon.

**Figure 8:**
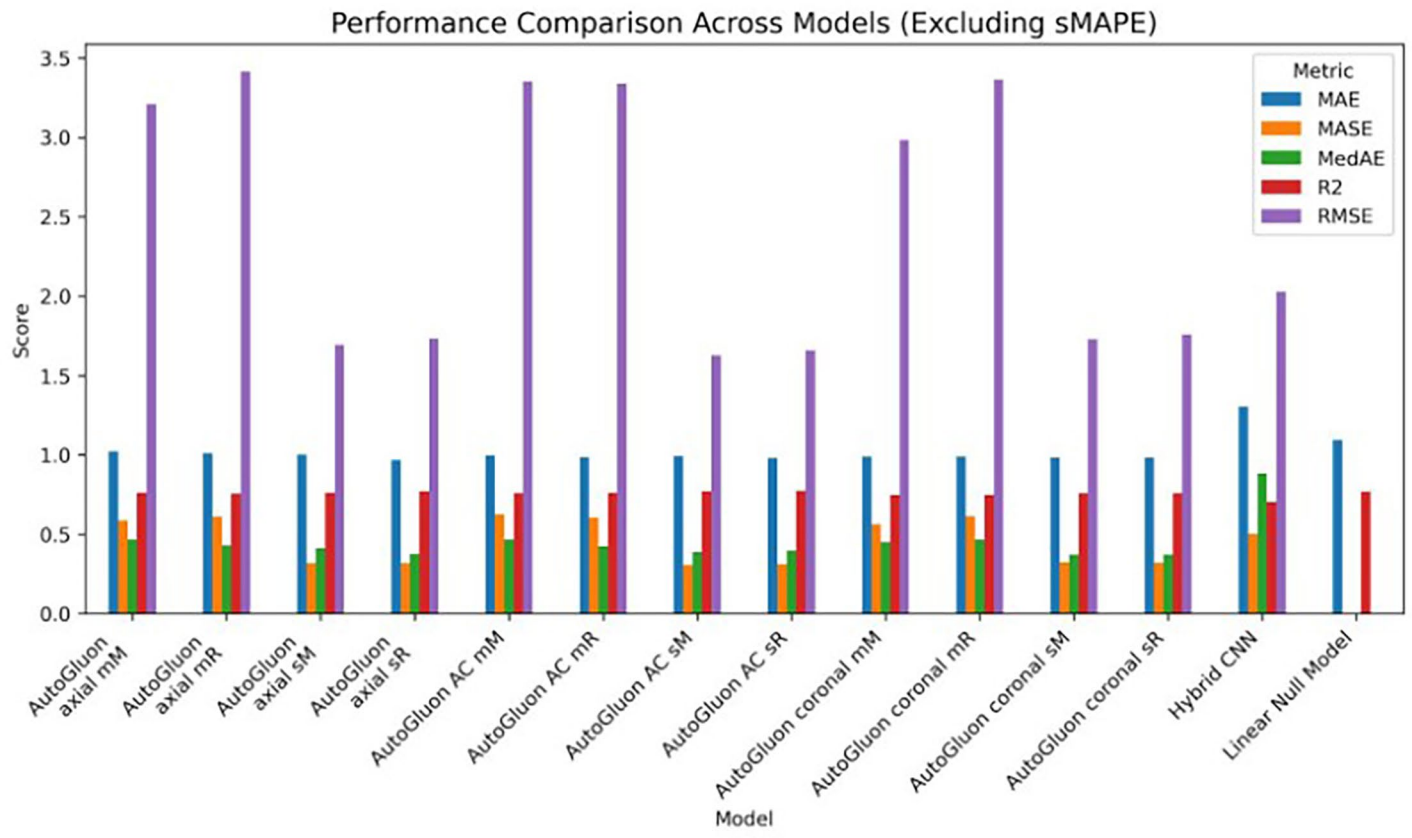
Bar chart of average performance across five folds for all models (excluding sMAPE). The grouped bar chart depicts mean values of MAE, RMSE, R^2^, MedAE, and MASE for each model. Note that for error metrics (MAE, RMSE, MedAE, MASE) lower values indicate better performance, while for R^2^ higher values indicate better performance.

**Figure 9:**
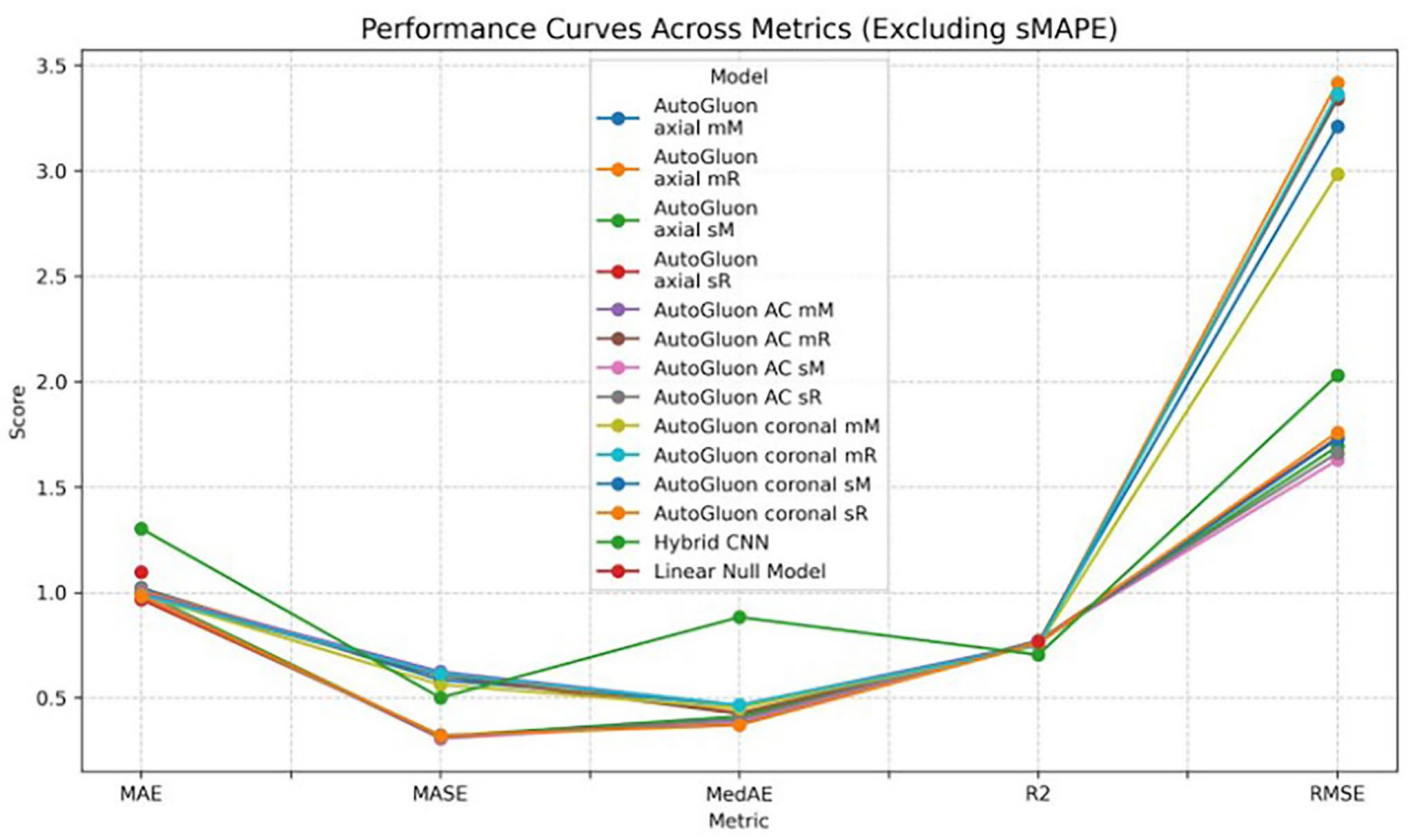
Performance curves across evaluation metrics for all models (excluding sMAPE). Average results (MAE, RMSE, R^2^, MedAE, and MASE) are shown as continuous curves, highlighting differences in model behavior across metrics. Note that for error metrics (MAE, RMSE, MedAE, MASE) lower values indicate better performance, while for R^2^ higher values indicate better performance.

**Figure 10:**
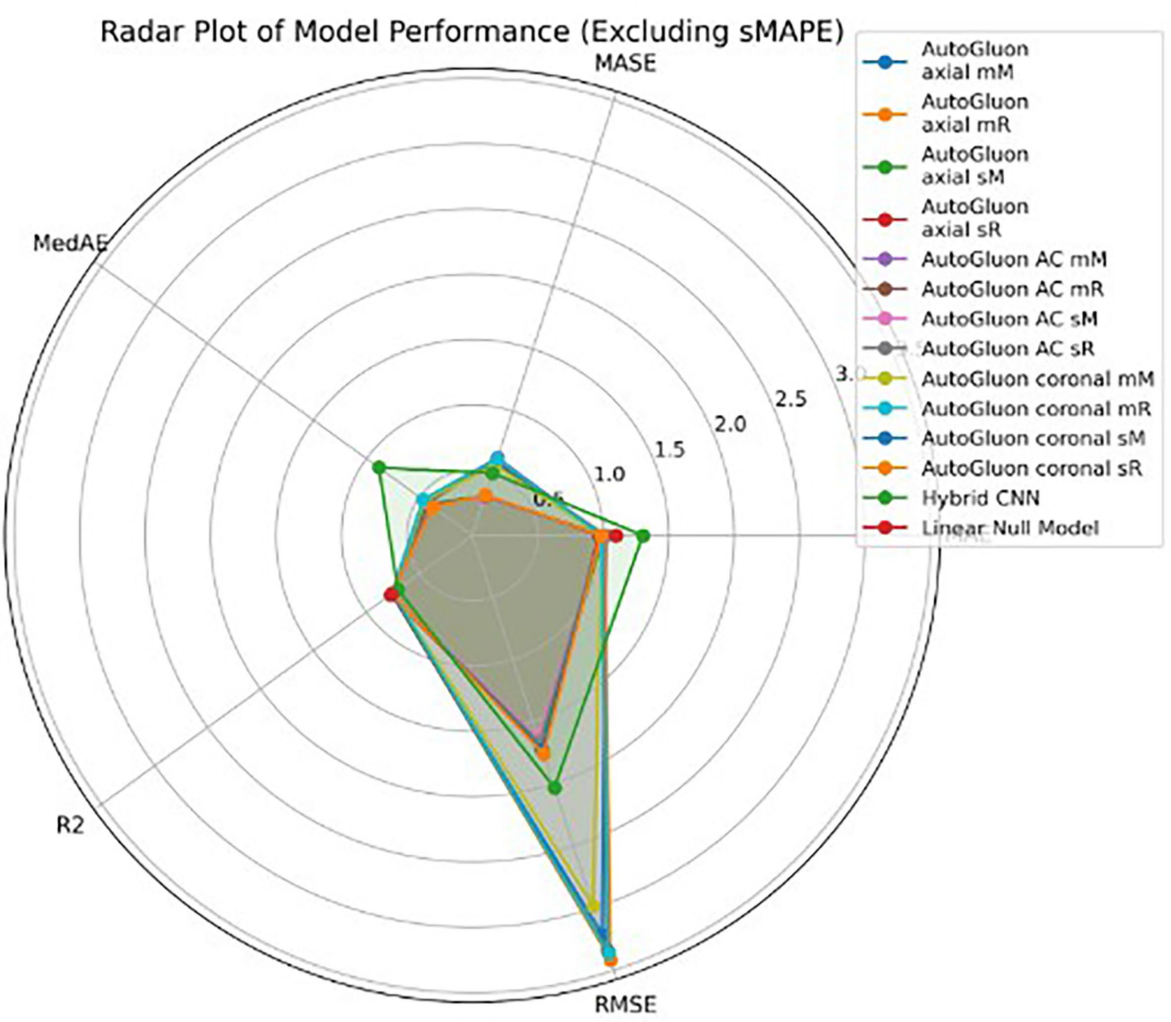
Radar plot of model performance across evaluation metrics (excluding sMAPE). The radar chart summarizes mean values of MAE, RMSE, R^2^, MedAE, and MASE across folds. Note that for error metrics (MAE, RMSE, MedAE, MASE) lower values indicate better performance, while for R^2^ higher values indicate better performance.

As an ablation study, a simple general linear model was constructed to estimate the predictive capacity when using only the tabular covariates to estimate the outcome across the five folds. This model does not take into account any imaging information at all. Comparing the results to AutoGluon, it shows that adding imaging features helps to improve the accuracy of the prediction. The performance of AutoGluon’s MultimodalFusionMLP model did not greatly improve when using a combination of multiple T1-weighted MRI middle coronal and axial slices and demographic features. The same training configurations were used as the previous case - two different validation approaches and two different scaling methods, assessed through 5-fold cross validation. Using standard scaling, the model achieved an average R^2^ score of 0.773 and MAE of 0.981. Using min-max scaling, the model achieved an average R^2^ score of 0.759 and MAE of 0.999. These results are consistent with those when only a single slice was used as the image input. Table 2 includes the performance of all experiments. Figures 8–10 show the performance of all models across the five folds.

## 4 Discussion

We do not assume that the hybrid CNN will be the best possible multimodal model under current sample size, but rather as a probe for when volumetric MRI contributes meaningful prognostic value. In this dataset, tabular features explain a large portion of variance, leading AutoML fusion to outperform full 3D fusion. The CNN’s performance suggests that volumetric features may only help substantially when imaging contains additional, non-redundant prognostic signals or when the dataset size is larger, to support high-dimensional image learning. The observed gap does not undermine the utility of volumetric fusion, but it reveals when simpler multimodal pipelines may be preferable. Our hybrid deep learning model demonstrated strong predictive performance in forecasting sobCDR progression, as evidenced in the 5-fold cross-validation. The model achieved an average MAE of 1.303, indicating high accuracy in estimating cognitive decline, with consistent performance across folds (MAE range: 1.231–1.414). Additionally, the R^2^ score averaged 0.704, reflecting a strong correlation between predicted and actual clinical scores. These results highlight the model’s robustness and reliability across multiple cohorts and datasets, suggesting its promise for real-world clinical applications. Minor variations were observed across folds, but the model consistently captured predictors needed to forecast disease progression. AutoGluon’s MultimodalFusionMLP model performed well for predicting clinical decline using neuroimaging and demographic data. The consistent R^2^ values around 0.75–0.77 across different validation approaches and scaling methods suggest that the model captures around 76% of the variance in clinical outcomes, which is promising for this complex medical outcome. Performance did not further improve when using multiple slices versus using a single brain MRI slice as the image input. AutoGluon outperformed the Hybrid CNN across most folds. Our findings indicate that tabular features already perform well for prognostic predictions when they capture baseline disease severity, and multimodal CNNs may offer benefits only when sufficiently large datasets or additional imaging modalities are available to overcome the high dimensionality of 3D volumes. Baseline disease severity is a strong predictor in progressive conditions such as Alzheimer’s disease, especially when we lack treatments to slow progression. Without disease modifying treatments, disease tends to progress more rapidly in those with clear signs of disease already. In addition, AutoML-based fusion can capitalize on structured data, such as baseline clinical and demographic data, making it effective in clinical settings where rich baseline phenotyping is available. To avoid the ambiguity of fold-to-fold fluctuations, we directly compared model performance across identical cross-validation splits using paired t-tests. AutoGluon showed a statistically significant improvement over the Hybrid CNN in terms of both prediction error (MAE: *p* = 0.0003, chance of Type I error ≈ 0.03%) and explained variance (R^2^: *p* = 0.0076, chance of Type I error ≈ 0.76%). These results confirm that AutoGluon’s apparent superiority is not attributable to random fold-level variability. The tabular covariates carry substantial predictive signals and AutoGluon is particularly well-suited to exploit such structured data through automated architecture search and ensembling. In contrast, our hybrid CNN is more data-hungry due to its 3D volumetric imaging branch, which may require larger training datasets to consistently leverage image features. Thus, AutoML provides a strong baseline in tabular-dominant prediction settings, whereas the advantages of hybrid 3D CNNs may emerge more clearly as training cohorts scale.

A direct dimensionality-matched comparison between AutoGluon and the hybrid CNN is not currently feasible, as AutoGluon does not support 3D input encoders. Moreover, reducing the CNN to 2D slices would remove the volumetric inductive bias (Gupta et al., 2023; Dhinagar et al., 2023) being evaluated and fundamentally change the model’s purpose. Therefore, the comparison should be interpreted not as a contest between two identical architectures, but as a benchmarking of two realistic multimodal strategies: a lightweight AutoML fusion with reduced imaging, versus a full-volumetric fusion model designed to exploit global 3D anatomy. Relative performance may depend on modality dominance, dataset size, and imaging richness rather than architecture alone.

Several key observations emerge from our analysis:

(1) Scaling method: standard scaling and MinMax scaling produced comparable results, with standard scaling yielding marginally better performance (R^2^ = 0.771 vs. 0.757 under R^2^ validation). This suggests that the model is robust to the choice of feature scaling method, though standard scaling might offer a slight advantage in capturing the underlying relationships in the data. (2) Validation metric: the similar performance on MSE and R^2^ validation metrics (MAE ≈ 1.0 across configurations) indicates that the models’ predictive capability is stable regardless of the optimization criterion. This robustness is valuable in clinical applications where consistency across different evaluation methods is crucial. (3) Cross-validation stability: the relatively small variations in performance across folds (R^2^ standard deviation ≈ 0.013–0.027) suggest that the model generalizes well across different subsets of the data. This stability is needed for clinical applications, where reliable performance across diverse patient populations is essential. (4) Clinical implications: the achieved MAE of around 1 point in predicting future decline represents a clinically meaningful level of accuracy, considering the scale and complexity of the factors that affect future clinical decline. This level of accuracy may potentially support clinical decision making. Realistically, the lower bound on prediction error depends on the reliability of the clinical test itself, as the test does not have perfect test–retest reliability when administered by different raters, or on different days. Prior work on the sobCDR score suggests that test–retest reliability is good (intra-class correlation coefficient [ICC] = 0.83) (McDougall et al., 2021); in one study, 24 human raters independently scored the CDR using four videotaped interviews (Rockwood et al., 2000), and there was moderate to high overall interrater reliability (kappa statistic: 0.62), despite some difficulties in reliably assessing early dementia. Even so, a more recent study (Dauphinot et al., 2024) of 139 patients (age: 80.1 ± 6SD, 72% with dementia) reported a much higher ICC of 0.95 (95% CI, 0.93–0.97) for the sobCDR score assessed face-to-face and with all the information available in the patient’s medical record. The mean difference between the sobCDR score assessed face-to-face and with the medical record was 0.098 ± 1.036. These findings underscore that the inherent variability of the sobCDR, which typically is on the order of ~1 point across raters and sessions, effectively defines the achievable limits of predictive model performance. In this context, the practical significance of an MAE of ~1 point should be interpreted relative to clinical thresholds. Prior clinical studies have established that a change of 1–2 points on the CDR-SB over 18–24 months represents a minimal clinically important difference (MCID) and corresponds to clinically meaningful worsening. Borland et al. (2022) demonstrated in the BioFINDER cohort that a ≥ 1 point increase in CDR-SB is clinically relevant in preclinical and prodromal stages. Andrews et al. (2019) analyzed large AD clinical trial datasets and estimated MCIDs between 0.74 and 1.19 points over ~18 months. Petersen et al. (2023) further summarized that a 1–2 point change is widely recognized in both trial and regulatory contexts as a benchmark threshold for meaningful decline. Taken together, these findings indicate that the predictive precision achieved here (MAE ≈ ±1 point) is comparable to the inherent measurement variability of the sobCDR itself and lies within the range of changes considered clinically significant, suggesting potential utility for patient stratification, longitudinal monitoring, and clinical trial recruitment.

An important consideration in this study is the choice to formulate sobCDR prediction as a regression problem rather than classification. Although sobCDR values increment in 0.5-point steps, they are derived from summing ratings across multiple domains and are interpreted in clinical research and trials as quasi-continuous. Modeling sobCDR as a continuous outcome is therefore more consistent with clinical usage, where mean changes in the scale, rather than categorical thresholds, are the basis for interpreting disease progression and treatment effects. From a methodological perspective, regression modeling leverages the full range of observed sobCDR values, including fractional increments, avoiding information loss and allowing sensitivity to subtle but clinically significant shifts. In contrast, discretizing sobCDR into categories for classification would impose arbitrary boundaries and obscure the magnitude of error, which is essential when determining whether predicted changes exceed MCID thresholds. Regression metrics such as MAE, RMSE, and R^2^ quantify prediction error on the same continuous scale used in clinical interpretation, enabling a clearer link between model outputs and their practical implications. In summary, while sobCDR values are technically discrete, the regression formulation adopted here aligns with both clinical convention and methodological precedent in dementia prognostics. This framing ensures that predicted scores can be directly compared against clinically meaningful thresholds, thereby enhancing translational relevance.

For the hybrid CNN, extending voxel-level attribution methods (e.g., Grad-CAM) to the fused network is non-trivial, as image and tabular embeddings are concatenated into a joint latent representation before prediction, making it difficult to isolate saliency uniquely attributable to MRI. A promising direction is to apply Grad-CAM or Layerwise Relevance Propagation (LRP) to the image branch as a surrogate explainer, quantifying which brain regions the CNN attends to before fusion. In parallel, feature attribution tools such as SHAP or Integrated Gradients could quantify the relative contribution of clinical variables in the tabular branch, making it possible to compare imaging versus non-imaging importance. More advanced fusion mechanisms -including cross-modal attention (Song et al., 2022), gated fusion (Li et al., 2025), and mixture-of-experts layers (Han et al., 2024) - may provide interpretable attribution scores that reveal when imaging meaningfully influences predictions. Such architectures would allow MRI saliency to be weighted by clinical state, which may be useful in prognostic settings where baseline severity is strongly predictive. Therefore, future work will evaluate attention-based interpretability, surrogate voxel-level attribution, and cross-modal importance metrics to better understand how and when MRI features impact predictive prognosis. Ultimately, explainability methods may help determine where imaging contributes to a model, or when it contributes minimally - and this could guide when multimodal imaging is necessary versus redundant.

## 5 Limitations

One limitation of our current implementation is the use of a very small batch size (n = 2) for training the hybrid CNN, imposed by GPU memory constraints when using 3D volumetric input data. While we employed batch normalization, early stopping, and cross-validation to stabilize training, future work leveraging gradient accumulation, mixed-precision training, and higher-memory hardware may allow larger batch sizes and may improve feature learning. While the models showed robustness across different validation folds, future work will attempt to further improve accuracy by incorporating additional data types, such as diffusion MRI or PET data, genome-wide genetics, and blood-derived proteomic markers, as well as longitudinal clinical assessments. While our preprocessing pipeline reduces scanner-related variability, residual batch effects across cohorts may still influence results. Future work will investigate advanced harmonization approaches such as ComBat-GAM or deep-learning–based domain adaptation to further control site-specific biases and improve generalizability. Additionally, advanced techniques such as self-supervised learning and transformer-based architectures that can handle missing data (Shirkavand et al., 2023) may further enhance performance, and may also help to predict decline in specific subdomains of cognitive performance. Interpretability remains a key priority for clinical deployment. For the hybrid CNN, however, extending Grad-CAM to the fused multimodal network is non-trivial, as the concatenation of image and tabular features complicates voxel-level attribution. Similarly, AutoGluon does not natively expose interpretability tools, but future work will examine surrogate explainability methods and attention-based multimodal fusion, which may allow more transparent cross-modal attributions. A separate held-out test set was not used as we had only a limited number of participants meeting the strict clinical and imaging inclusion requirements across all cohorts. Although stratified five-fold cross-validation is widely used to estimate generalization in neuroimaging-based prognostic models, it does not fully replace an independent test set. Future work will include external validation on additional independent cohorts as they become available, to further assess out-of-distribution generalization. Validating the model on new and more diverse clinical datasets will be crucial for assessing real-world applicability and potential integration into clinical decision-making frameworks.

## 6 Conclusion

Our results show the potential of multimodal deep learning for accurately forecasting cognitive decline–a line of work that could aid in early diagnosis and personalized treatment planning for neurodegenerative diseases. Two clear priorities for future work emerge from this study. First, expanding modality coverage by integrating complementary data types such as diffusion MRI, other types of T2-weighted imaging, PET, genomics, and blood-based proteomic markers will help to capture a broader spectrum of disease biology. Second, refining model architecture through self-supervised pretraining, transformer-based fusion mechanisms, and advanced explainability methods will allow more effective use of heterogeneous data while maintaining clinical interpretability. Together, these directions will enhance predictive accuracy and translational value for real-world deployment. Overall, our study illustrates how integrating artificial intelligence methods such as multimodal deep learning and AutoML frameworks into hospital workflows could enhance early detection and prognosis of cognitive decline, contributing to the broader digital transformation of healthcare systems.

## Data Availability

Publicly available datasets were analyzed in this study. This data can be found here: the datasets analyzed for this study are publicly available to qualified investigators, and may be found at: https://adni.loni.usc.edu, https://naccdata.org, and https://www.oasis-brains.org.
